# Increased *O*-GlcNAcylation of Drp1 by amyloid-beta promotes mitochondrial fission and dysfunction in neuronal cells

**DOI:** 10.1186/s13041-020-00727-w

**Published:** 2021-01-09

**Authors:** So Jung Park, Ji-Eun Bae, Doo Sin Jo, Joon Bum Kim, Na Yeon Park, Jianguo Fang, Yong-Keun Jung, Dong Gyu Jo, Dong-Hyung Cho

**Affiliations:** 1 Department of Medical Genetics, Cambridge Institute for Medical Research, University of Cambridge, Cambridge, CB2 0XY UK; 2 Brain Science and Engineering Institute, Graduate School of Life Science, Kyungpook National University, Daegu, 41566 Republic of Korea; 3 Graduate School of Life Science, BK21 FOUR KNU Creative BioResearch Group, Kyungpook National University, 80 Daehakro Bukgu, Daegu, 41566 Republic of Korea; 4 State Key Laboratory of Applied Organic Chemistry, College of Chemistry and Chemical Engineering, Lanzhou University, Lanzhou, 730000 People’s Republic of China; 5 School of Biological Sciences, Seoul National University, Seoul, 08826 Republic of Korea; 6 School of Pharmacy, Sungkyunkwan University, Suwon, 16419 Republic of Korea

**Keywords:** Drp1, *O*-GlcNAcylation, Mitochondrial fission, Amyloid-beta, Alzheimer’s disease

## Abstract

As a dynamic organelle, mitochondria continuously fuse and divide with adjacent mitochondria. Imbalance in mitochondria dynamics leads to their dysfunction, which implicated in neurodegenerative diseases. However, how mitochondria alteration and glucose defect contribute to pathogenesis of Alzheimer’s disease (AD) is still largely unknown. Dynamin‐related protein 1 (Drp1) is an essential regulator for mitochondria fission. Among various posttranslational modifications, *O*-GlcNAcylation plays a role as a sensor for nutrient and oxidative stress. In this study, we identified that Drp1 is regulated by *O*-GlcNAcylation in AD models. Treatment of Aβ as well as PugNAc resulted in mitochondrial fragmentation in neuronal cells. Moreover, we found that AD mice brain exhibits an upregulated Drp1 *O*-GlcNAcylation. However, depletion of OGT inhibited Drp1 *O*-GlcNAcylation in Aβ-treated cells. In addition, overexpression of *O*-GlcNAc defective Drp1 mutant (T585A and T586A) decreased Drp1 *O*-GlcNAcylation and Aβ-induced mitochondria fragmentation. Taken together, these finding suggest that Aβ regulates mitochondrial fission by increasing *O*-GlcNAcylation of Drp1.

Mitochondria are highly dynamic organelles that continuously undergo fission and fusion with adjacent mitochondria. Therefore, disruption of the balance between these two processes results in mitochondrial dysfunction and aberrations in physiological neuronal functions, which are linked with the pathogenesis of various neurodegenerative diseases [1]. Dynamin related protein 1 (Drp1), a GTPase, is a critical regulator of mitochondria and peroxisome fission [2]. Several posttranslational modifications on Drp1 such as phosphorylation and *S*-nitrosylation control mitochondrial dynamics by regulating its GTPase activity [3, 4]. *O*-linked β-*N*-acetylglucosamine (*O*-GlcNAc) can dynamically modify target proteins by *O*-GlcNAcylation, which attaches *O*-GlcNAc to proteins. *O*-GlcNAcylation is controlled by two enzymes, *O*-GlcNActransferase (OGT) and *N*-acetyl-glucosaminidase (OGA) [5]. Mitochondrial dysfunction by amyloid-beta_1–42_ (Aβ) is an early and prominent feature of Alzheimer’s disease (AD). A number of *O*-GlcNAcylated proteins are associated with the pathology of neurodegenerative diseases. Although it was reported that cardiac Drp1 is undergoes more *O*-GlcNAcylation in type 2 diabetic mice [6], the link between Drp1 *O*-GlcNAcylation and AD pathology remains largely unknown.

To examine the *O*-GlcNAcylation of Drp1 in AD models, primary cultured neurons were treated with either Aβ or PugNAc, an OGA inhibitor, with consequent increase of *O*-GlcNAcylation. We found that both Aβ and PugNAc treatment induced mitochondrial fragmentation in primary cultured neurons, suggesting that *O*-GlcNAcylation influenced mitochondria fragmentation (Fig. 1a). As WGA-conjugated-agarose beads bind to *O-*GlcNAc-modified proteins [7], we employed a WGA antibody-conjugated agarose beads to confirm whether Drp1 is target for *O*-GlcNAcylation in Aβ-treated cells. As shown Fig. 1b, c, notably, Aβ treatment strongly promoted *O*-GlcNAcylation of Drp1 in both SH-SY5Y neuroblastoma cells and primary cultured neurons (Fig. 1b, c). As mitochondrial fission is increased in AD brain, we wondered whether the level of Drp1 *O*-GlcNAcylation is enhanced in the brain tissues of mice AD model (5 × FAD transgenic mice) that overexpress human APP and PSEN1 transgenes with a total of five AD-linked mutations [8]. Previously, it was reported that *O*-GlcNAcylation of nicastrin is elevated in brain of 5 × FAD mice [9]. Consistently, we found that Drp1 *O*-GlcNAcylation and nicastrin are upregulated in 5 × FAD mice brain compared to that of wild type mice brain (Fig. 1d).

**Fig. 1:**
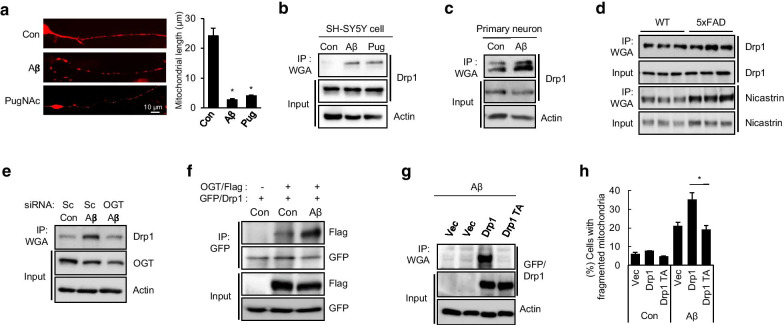
Aβ induces mitochondria fragmentation by promoting Drp1 *O*-GlcNAcylation in neuronal cells. **a** Primary cultured neuron were exposed to control (Con), Aβ (10 µM), or PugNAc (100 µM). Mitochondria were stained with mitotracker-Red. The mean mitochondrial length was determined from images of multiple cells obtained by microscopy. The bar graph indicates means ± SEM. *p < 0.01. The scale bar indicates 10 µm. **b** Drp1 *O*-GlcNAcylation in response to either Aβ (10 µM) or PugNAc (100 µM) in SH-SY5Y. Cell lysates were immunoprecipitated (IP) with anti-WGA-agarose antibody and immunoblotted with anti-Drp1 antibody. **c** Drp1 *O*-GlcNAcylation by Aβ treatment in primary neuron. **d** Enhanced Drp1 *O*-GlcNAcylation in brain tissue of 5 × FAD. Mice brain lysates from wild-type and 5 × FAD were assessed by IP using anti-WGA-agarose antibody and confirmed with anti-Drp1 and anti-Nicastrin antibodies. **e** Depletion of OGT reduced *O*-GlcNAcylation of Drp1. SH-SY5Y cells transfected with scrambled siRNA (Sc) or siRNA for OGT were treated with Aβ (10 µM) and the cell lysates were assessed by IP with anti-WGA-agarose antibody and immunoblotted with anti-Drp1. **f** Interaction between Drp1 and OGT. SH-SY5Y expressing OGT-flag and GFP-Drp1 were treated with Aβ (10 µM) and then assessed with IP (anti-GFP antibody) and immunoblotted with anti-Flag and anti-GFP antibodies. **g** *O*-GlcNAcylation of mutant Drp1. SH-SY5Y cells expressing empty vector (Vec), GFP-Drp1 (Drp1), or GFP-Drp1 T585A/586A mutant (Drp1 TA) were treated with Aβ (10 µM). The cell lysates were assessed by IP with anti-WGA-agarose antibody and immunoblotted with anti-GFP-antibody. **h** Mitochondrial fragmentation with Drp1 mutant. SH-SY5Y cells expressing empty vector (Vec), GFP-Drp1 (Drp1), or GFP-Drp1 T585A/586A mutant (Drp1 TA) were treated with Aβ (10 µM). Cells with fragmented mitochondria were counted under a microscopy. The bar graph indicates means ± SEM. *p < 0.01

To further investigate how Aβ regulates Drp1 *O*-GlcNAcylation, we examined the role of OGT, which attach *O*-GlcNAc to target protein in SH-SY5Y cells. As expected, depletion of OGT by RNA interference reduced Drp1 *O*-GlcNAcylation in Aβ-treated cells (Fig. 1e). We then confirmed the possibility of an interaction between OGT and Drp1. SH-SY5Y cells overexpressing GFP-tagged Drp1 and Flag-tagged-OGT were prepared. An immunoprecipitation assay showed that Drp1 interacts with OGT and that interaction was much more enhanced by Aβ exposure compared with that of control cells (Fig. 1f). Previously, Gawlowski et al. suggested that the residues Thr-585 and Thr-586 on Drp1 are putative targets for *O*-GlcNAc [6]. To confirm the presence of Drp1 *O*-GlcNAcylation residue, we generated an *O*-GlcNAc-defective Drp1 mutant (T585A and T586A/Drp1 TA). SH-SY5Y cells expressing GFP fused wild type Drp1 or GFP-fused Drp1 TA were analyzed by immunoprecipitation assay. According to the previous results, *O*-GlcNAcylation of Drp1 was did not occur in the Drp1 TA mutant in Aβ-treated cells (Fig. 1g). To clarify the effect of *O*-GlcNAcylation on Drp1 activity, we monitored mitochondrial morphology in Aβ-treated cells. As shown in Fig. 1h, ectopic expression of Drp1 promoted mitochondrial fragmentation in Aβ-treated cells. However, overexpression of Drp1 TA mutant did not enhance mitochondrial fission in Aβ-treated cells (Fig. 1h). Taken together, these results suggest that Thr-585 and Thr-586 residues on Drp1 are *O*-GlcNAcylated and contribute to mitochondrial fragmentation in Aβ-exposed cells.

Overstimulation of Drp1 results in excessive mitochondrial fragmentation and previous studies have reported that Drp1 activity is regulated by several post-translational modifications, such as phosphorylation, *S*-nitrosylation, SUMOylation, ubiquitination, and *O*-GlcNacylation [1, 4]. Among the modifications, we highlighted that *S*-nitrosylation of Drp1 at Cys644 caused mitochondrial fragmentation by increasing its activity in brain neurons from AD and Huntington’s disease patients [3, 10]. In this study, in addition, we suggest that Aβ can induce mitochondrial fission and dysfunction by promoting *O*-GlcNAcylation of Drp1 in AD models. These findings suggest that Aβ enhances Drp1 activity and causes excessive mitochondrial fission in various way, leading to mitochondrial dysfunction and neuronal death in AD progression. *O*-GlcNAcylation and phosphorylation share medication target residues and their interplay is highly complex and comprehensive [5]. Therefore, further studies on the implication of *O*-GlcNAcylation and phosphorylation on regulation of Drp1 activity will be helpful to provide insight on the role of mitochondrial dynamics in AD pathogenesis.

## Data Availability

All data generated or analyzed during this study are included in this published article.

## Abbreviations

AD: Alzheimer’s disease
Drp1: Dynamin-related protein 1
Aβ: Amyloid-beta
